# The Etiology, Incidence, and Impact of Preservation Fluid Contamination during Liver Transplantation

**DOI:** 10.1371/journal.pone.0160701

**Published:** 2016-08-11

**Authors:** Isabel Oriol, Laura Lladó, Marina Vila, Carme Baliellas, Fe Tubau, Núria Sabé, Joan Fabregat, Jordi Carratalà

**Affiliations:** 1 Department of Infectious Diseases, Hospital de Bellvitge, Institut d’Investigació Biomèdica de Bellvitge, University of Barcelona, L’Hospitalet de Llobregat, Barcelona, Spain; 2 Liver Transplant Unit, Hospital de Bellvitge, Institut d’Investigació Biomèdica de Bellvitge, University of Barcelona, L’Hospitalet de Llobregat, Barcelona, Spain; 3 Department of Microbiology, Hospital de Bellvitge, Institut d’investigació Biomèdica de Bellvitge, University of Barcelona, l’Hospitalet de Llobregat, Barcelona, Spain; Istituto Mediterraneo per i Trapianti e Terapie ad Alta Specializzazione, ITALY

## Abstract

The role of contaminated preservation fluid in the development of infection after liver transplantation has not been fully elucidated. To assess the incidence and etiology of contaminated preservation fluid and determine its impact on the subsequent development of infection after liver transplantation, we prospectively studied 50 consecutive liver transplants, and cultured the following samples in each instance: preservation fluid (immediately before and at the end of the back-table procedure, and just before implantation), blood, and bile from the donor, and ascitic fluid from the recipient. When any culture was positive, blood cultures were obtained and targeted antimicrobial therapy was started. We found that the incidence of contaminated preservation fluid was 92% (46 of 50 cases of liver transplantation per year), but only 28% (14/50) were contaminated by recognized pathogens. Blood and bile cultures from the donor were positive in 28% and 6% respectively, whereas ascitic fluid was positive in 22%. The most frequently isolated microorganisms were *coagulase-negative staphylococci*. In nine cases, the microorganisms isolated from the preservation fluid concurred with those grown from the donor blood cultures, and in one case, the isolate matched with the one obtained from bile culture. No liver transplant recipient developed an infection due to the transmission of an organism isolated from the preservation fluid. Our findings indicate that contamination of the preservation fluid is frequent in liver transplantation, and it is mainly caused by saprophytic skin flora. Transmission of infection is low, particularly among those recipients given targeted antimicrobial treatment for organisms isolated in the preservation fluid.

## Introduction

Liver transplantation has become the definitive treatment of several end-stage liver diseases [1–3]. In recent years, with the growing disparity between organ availability and the number of candidates for liver transplantation, most centers have extended the acceptance criteria for donors [4,5]. However, this strategy increases the risk of bacterial, viral, and fungal infections after transplantation [6,7], making post-transplant infection a leading cause of in-hospital mortality among liver transplant recipients [8–10].

Contamination of preservation fluid has been identified as a potential source of post-transplant infection [6,11], although the reported incidence ranges from as low as 9.5% to as high as 98.4% [7,12–14]. The frequency of infection transmitted via the preservation fluid varies depending on the type of the study [6,13]. Moreover, related case-fatality rates have been reported to exceed 50%, depending on the causative organisms [13]. However, our current understanding of the contamination of preservation fluid and subsequent recipient infection derives to a large extent from retrospective studies in which only a few fluids from patients were examined.

To detect allograft contamination and improve the early diagnosis and approach of infection-related complications, some transplant centers now routinely take intra-operative cultures of organ preservation fluid. However, evidence supporting this practice is scarce, and little is known about the most appropriate management strategy for liver recipients with confirmed preservation fluid contamination. In this study, we aimed to clarify the incidence and etiology of preservation fluid contamination in liver transplantation to determine the role of this contamination in the subsequent development of infection.

## Materials and Methods

We conducted a prospective observational study at a tertiary university referral hospital for adults in Barcelona, Spain, where some 50 liver transplants are performed annually. For this study, we included all liver transplants from September 2012 to August 2013, collecting demographic and medical information from the donors and the recipients (including immunosuppressive treatment, occurrence of acute allograft rejection, infections, clinical features, microbiologic studies, and outcomes) as well as the operative data. All data were carefully recorded in a database. Differences in donor, baseline, and surgical characteristics of those undergoing liver transplantation were evaluated.

None of the transplant donors were from a vulnerable population and all donors or next of kin provided written informed consent that was freely given. Written informed consent was considered not necessary for the study, as it was an analysis of our usual everyday work. The data of the patients were anonymized for the purposes of this analysis. The confidential information of the patients was protected according national normative. This manuscript was approved for its publication by the Clinical Research Ethics Committee of Bellvitge University Hospital.

The microbiology reports for all the positive cultures (blood and preservation fluid) were reviewed daily by the infectious disease team, and patients were then followed up by an infectious disease physician. When pathogens were isolated from donor or preservation fluids, blood cultures were taken from the recipient and targeted antimicrobial therapy was prescribed. However, treatment was stopped if no clinical signs of infection developed or if cultures from the recipient were negative.

Grafts were routinely preserved in Celsior solution at 4°C. The liver was placed in a sterile plastic double-bag and transported in a third non-sterile bag. Back-table procedures were performed in a separate dedicated room near to where the organ retrieval was performed.

In order to detect the exact time when the PF is contaminated, and to understand which is the main mechanism involved in the contamination, three samples of preservation fluid were routinely obtained under sterile conditions (immediately before the back-table procedure, at the end of the back-table procedure, and just before implantation). Under sterile conditions, we also systematically obtained blood and bile samples from donors and ascites samples from recipients.

Preservation fluid contamination during the back-table procedure was defined as isolation of any organism from the sample obtained after the back-table procedure, but not in the sample recovered before the back-table procedure. Contamination of the preservation fluid during liver transplantation was defined as isolation of any organism from the sample obtained just before the graft implantation, but not recovered from the sample obtained before the back-table procedure.

Infections were defined according to the CDC/NHSN guidelines [15]. In case of infection after liver transplantation, the source of infection was determined by clinical criteria and isolation of any organism from a clinically significant site of infection. When no sites were identified, infection was considered to be from a primary or unknown source.

Prior transplantation referred to re-transplantation of the same organ. Empirical antibiotic therapy was considered inadequate if the treatment regimen did not include at least one antibiotic with in vitro activity against the infecting microorganism. Shock was defined as a systolic blood pressure of less than 90 mmHg, which was unresponsive to fluid treatment or required vasoactive drug therapy [16]. Acute allograft rejection was diagnosed only when proven by biopsy. The overall case-fatality rate was defined as death by any cause within the first 30 days of the onset of infection.

All patients received perioperative antibacterial prophylaxis with teicoplanin plus aztreonam for up to 24 hours after transplantation. Antifungal prophylaxis was given to high-risk recipients according to current recommendations [17]. Prophylaxis against *Pneumocystis jiroveci* infection was with trimethoprim–sulfamethoxazole given as a single dose, three times a week, for the first 6 months after transplantation. Prophylaxis against cytomegalovirus (CMV) was given according to the current guidelines [18].

Samples were processed by the BACTEC 9240 method (Becton-Dickinson Microbiology Systems, Sparks, MD). The inoculated bottles were incubated for five days at 35°C before being discharged. Microbial identification was performed using commercially available panels (MicroScan [Siemens; West Sacramento, United States] or Vitek [Biomérieux; Marcy-L’Étoile, France]), by standard biochemical and/or enzymatic test, or by matrix-assisted laser desorption ionization (MALDI-TOF; Bruker Daltonik; Bremen, Germany). The Clinical and Laboratory Standards (CLSI) criteria were used to define susceptibility or resistance to antimicrobial agents. Antibiotic susceptibility was tested using the micro-dilution method, following CLSI guidelines [19].

The microbiologic results of the preservation fluid culture were compared to the results of samples from the donor and the recipient. The criteria for microorganism matching were based on species identification and susceptibility profiles.

The statistical analysis was performed using PASW Statistics for Windows, Version 18.0 (SPSS, Inc., Chicago, IL, USA). Categorical variables were characterized by percentages and compared with chi-square tests, while continuous variables were expressed as means and standard deviations, or as medians and interquartile ranges (IQR), according to their distribution. Comparisons were made using the Mann- Whitney *U* test. All statistical tests were two-tailed and the threshold of statistical significance was <0.05.

## Results

We recorded the details of 50 consecutive liver transplantation among 47 patients during the study period. Table 1 shows the baseline characteristics for the liver donors and liver recipients, including the operation-related data. The median age of liver recipients was 57 (IQR 52–62) years, and 82% were male. The most frequent indication for liver transplantation was hepatocellular carcinoma, followed by chronic hepatitis C and alcoholic cirrhosis. The median age of donors was 63 (IQR 51–69) years, and 64% were male. Two were domino transplants, but the remaining forty-eight were deceased-donor transplants. Causes of death of donors were stroke (35), head trauma (8), cerebral anoxia (4), and other (1). The median time of ischemia was 377 (IQR 310–480) minutes, and the median time of surgery was 385 (IQR 344–450) minutes.

**Table 1:** Baseline liver donor and recipient characteristics, and operation features characteristics.

CHARACTERISTICS	N = 50
**Donor features**	
Age, median (IQR)	63 (51–69)
Weight (Kg), median (IQR)	80 (72–90)
Height (m), median (IQR)	1.7 (1.65–1.8)
Male sex, no. (%)	32 (64)
Heart disease, no. (%)	13 (26)
Length of ICU stay, median (IQR)	2 (1–6)
**Operative features**	
Cold ischemia time (minutes), median (IQR)	377 (310–480)
Length of surgery (minutes), median (IQR)	385 (344–450)
Red blood cell transfusion, no. (%)	29 (58)
Fresh-frozen plasma transfusion, no. (%)	15 (30)
Platelets transfusion, no. (%)	27 (54)
**Recipient features**	
Age, median (IQR)	57 (52–62)
Male sex, no. (%)	41 (82)
HTA pre-LT, no. (%)	13 (26)
DM pre-LT, no. (%)	9 (18)
MELD, median (IQR)	20 (15–25)
Length of ICU stay post-LT (days), median (IQR)	3 (2–4)
Length of hospitalization post-LT (days), median (IQR)	12 (9–20)
Infection during the first month post-LT, no. (%)	6 (12)
Overall case-fatality rate, no. (%)	1 (2)

IQR: Interquartile range; ICU: Intensive Care Unit; HTA: arterial hypertension; DM: Diabetes mellitus; LT: liver transplantation; MELD: Model of End-Stage Liver Disease

The microorganisms isolated are detailed in full in Table 2. All cultures of the preservation fluids collected before the back-table procedure were negative. One or more microorganisms were isolated in the preservation fluids collected just before implantation in 46 out of 50 transplants, representing an incidence rate of 92% for contamination of the preservation fluid. In eight cases the contamination was polymicrobial, and in 14 cases (28%) the isolates were considered pathogens. Blood samples and bile samples were positive in 14 (28%) and three donors (6%) respectively, and ascites samples were positive in 11 out of 50 recipients (22%); but only two isolates were considered pathogens in each setting. In nine cases, isolates from the preservation fluid were consistent with those isolated in blood cultures from the donor; these were *coagulase-negative staphylococci* in seven cases, and *streptococci viridans* and *candida* spp. in one case each. In another case, the preservation fluid isolate (*Enterobacter* spp.) matched that obtained from the bile culture. S1 File links information about each recipient and their donor, as well as the operative features characteristics and the microorganisms isolated in each case.

**Table 2:** Microorganisms isolated in the preservation fluid, blood, and bile samples from the donor and in the ascites samples from the recipients.

Microorganisms	PF-1^a^	PF -2^b^	PF -3^c^	Bile^d^	Blood^e^	Ascites^f^
**CNS**	-	8	32	1	10	7
***S*. *pneumoniae***	-	1	1	-	-	-
**MSSA**	-	-	5	-	-	-
***Enterobacter spp*.**	-	-	3	1	-	-
***Escherichia coli***	-	-	1	-	-	1
***Serratia spp*.**	-	-	1	-	-	-
***Enterococcus faecium***	-	-	2	-	-	1
***Enterococcus faecalis***	-	-	2	-	-	1
***Other streptococci spp*.**	-	1	6	-	1	-
***Haemophilus spp*.**	-	-	-	1	1	-
***Klebsiella pneumoniae***	-	-	-	-	1	-
***Bacillus spp*.**	-	-	1	1	-	1
***Corynebacterium spp*.**	-	1	1	-	-	-
***Candida spp*.**	-	-	1	-	1	-

CNS, Coagulase-negative staphylococci; MSSA, methicillin-sensitive *Staphylococcus aureus*; PF, preservation fluid; LT, Liver transplantation.

^a^ PF-1: Preservation fluid collected before the back-table procedure

^b^ PF-2: Preservation fluid at the end of the back-table procedure

^c^ PF-3: Preservation fluid collected just before implantation

^d^ Bile: bile sample from the donor

^e^ Blood: Blood sample from the donor

^f^ Ascites: Ascites sample from the recipient.

Table 3 shows data of liver recipients with contaminated preservation fluid before implantation regarding microorganisms involved, susceptibility to antibiotic prophylaxis, treatment and outcomes. The median duration of targeted antibiotic treatment, for those liver recipients with contaminated preservation fluid, was 6 (IQR 4.8–7.5) days.

**Table 3:** Liver recipients with contaminated preservation fluid before implantation: microorganisms involved, susceptibility to antimicrobial prophylaxis, treatment and outcomes.

Case	Organisms isolated in the PF	AAP	AP	Treatment	Days of treatment	Length of ICU stay (days)	LOS (days)	Infection^1^	Re-transplantation	Death
1	*Serratia* spp	Yes	Yes	Ertapenem	4	3	9	No	No	No
2	*Enterococcus faecalis Streptococcus viridans* CNS	Yes	Yes	Meropenem	6	2	26	Cholangitis and *E*.*faecium* bacteremia	Ischemic cholangitis	No
3	*Enterococcus faecium* CNS	Yes	Yes	Linezolid	15	2	12	No	No	No
4	*Enterobacter cloacae Bacillus cereus*	No	No	Ciprofloxacin	4	3	8	No	No	No
5	*Staphylococcus aureus*	Yes	No	Cefazolin	6	3	19	No	No	No
6	*Enterobacter cloacae Enterococcus faecalis*	No	No	Amoxicillin + Ciprofloxacin	7	3	12	No	No	No
7	*Staphylococcus aureus*	Yes	No	Teicoplanin/ Daptomycin	6	2	12	No	No	No
8	*Staphylococcus aureus Streptococcus sanguis*	Yes	No	Aztreonam + Teicoplanin	5	4	12	No	No	No
9	*Enterobacter aerogenes*	Yes	No	Ertapenem	10	8	34	No	No	No
10	*Staphylococcus aureus Enterococcus faecium*	Yes	No	Linezolid	9	10	23	No	No	No
11	*Streptococcus pneumoniae*	Yes	No	Ceftriaxone	4	2	7	No	No	No
12	*Staphylococcus aureus*	Yes	No	Daptomycin	5	2	8	No	No	No
13	*Escherichia coli*	Yes	Yes	Ceftriaxone	7	10	37	No	No	No
14	*Candida glabrata CNS*	No	No	Anidulafungin	7	2	10	No	No	No

Abbreviations: AAP, adequate antibiotic prophylaxis; AP, antifungal prophylaxis; CNS, Coagulase-negative staphylococci; ICU, Intensive Care Unit; LOS, Length of hospitalization post-LT; N, no; PF, preservation fluid; Y, yes

^1^ Infection during the first month post-LT

During the study period, only six recipients suffered infections during the first month after liver transplantation, although none of these was due to possible pathogen transmission from the donor to the recipient. Three recipients required re-transplantation for ischemic cholangitis, acute cellular graft rejection, and hepatic artery thrombosis. One recipient died because of an acute hepatic artery thrombosis.

Finally, we compared various donor and surgical features as well as the outcomes between liver recipients with and without the growth of pathogens in the preservation fluid culture collected just before implantation. Table 4 summarizes the results of the between-group comparison. No statistical differences were found.

**Table 4:** Comparative analysis by the culture result from the preservation fluid just before implantation.

Characteristics	Univariable analysis		
	Pathogens N = 14	Negative or SSF N = 36	p-value
**Donor features**			
Male sex	8 (57.1%)	24 (66.7%)	0.529
Age (years)	63.0 (53.8–73.3)	63.0 (51.0–69.0)	0.455
Diabetes mellitus	3 (23.1%)	10 (27.8%)	1
Heart disease	3 (21.4%)	10 (27.8%)	1
Length of ICU stay (Days)	1.0 (1.0–4.3)	3.0 (2.0–6.0)	0.033
**Operative features**			
Cold ischemia time (minutes)	411 (310–458)	360 (290–480)	0.582
Length of surgery (minutes)	360 (308–458)	390 (350–450)	0.380
**Outcomes**			
Length of ICU stay post-LT (days)	3.0 (2.0–5.0)	2.0 (2.0–4.0)	0.582
LOS post-LT (days)	12.0 (8.8–23.8)	11.0 (8.0–19.0)	0.871
Acute graft rejection	0	2 (5.6%)	1
Infection during the first month of LT	1(7.1%)	5 (13.9%)	0.663

ICU: Intensive Care Unit; MELD: Model of End-Stage Liver Disease; LOS: Length of stay; LT: liver transplantation; SSF, superficial saprophytic flora.

## Discussion

In this prospective study of 50 consecutive liver transplants, the incidence of preservation fluid contamination was 92% just before implantation. True pathogenic contamination accounted for 28%. Despite this high incidence, however, we did not identify any cases of infectious transmission.

Previous studies have reported a wide variability in the incidence of preservation fluid contamination [7,12–14], which may be due to the fact that some reports define contamination as the positive growth of any microorganism, while others require the isolation of known human pathogens [20,21]. In addition, the time of collection of samples during the process of liver transplantation, and the sensitivity of culture methods, differ among the published studies [6,22]. At present, little is known about the clinical value of collecting and culturing the preservation fluid, or about the appropriate management of recipients with positive cultures from preservation fluid samples.

Given that the prevalence of preservation fluid contamination was high, it is reasonable to speculate that the lack of infection transmission is related to the use of early, targeted antimicrobial treatment. However, we identified matches between isolates in the preservation fluid and isolates in the blood and bile cultures of donors, suggesting that this is a plausible route of infection transmission. It should be noted, however, that we did not perform a molecular epidemiology analysis, which would be necessary to confirm that these were truly the same microorganisms.

Few reports in the literature have shown the transmission of infection from donor to recipient through preservation fluid contamination [6,13,23,24]. When reported, the mortality rate in cases of infectious transmission is high [13,25]. Moreover, the scarce literature available suggests that transmission via the contamination of the preservation fluid is always related to known human pathogens. In the absence of data to the contrary, we should probably consider recipients at risk if these pathogens grow in preservation fluid cultures.

We collected preservation fluid samples at different times during the transplant procedure in an attempt to detect when preservation fluid was contaminated. In this regard, our findings concurred with those found by previous researchers who suggested that preservation fluid is most frequently contaminated during harvesting and manipulation in the back-table procedure [7]. Indeed, in the present study, all preservation fluid cultures before the back-table procedure were negative, while 92% of that collected just before implantation grew microorganisms. Moreover, the most frequently isolated microorganisms were superficial saprophytic flora, which could have occurred at any of a number of stages during the transplantation process, including the multiple-step procedures and the packaging of the liver after removal from the donor. Our data indicate that the most appropriate time to culture preservation fluids would be just before implantation and that taking other samples is probably of limited clinical value.

Among the strengths of this study are the prospective design and that we included a large number of different samples collected at different points during the transplant process, finding that contamination varies according to the time of samples. However, it should be acknowledged that our study has some limitations. For example, this was a single center study; in addition, due to the small size of our series, it was not possible to compare the differences between positive and negative cultures to identify risk factors for preservation fluid contamination.

## Conclusions

Preservation fluid contamination is frequent during liver transplantation, and it is mainly caused by superficial saprophytic flora. Transmission of infection is low among those recipients given targeted antimicrobial treatment for organisms isolated in the preservation fluid. However, prospective multicenter studies, which should include molecular epidemiological analyses, are required to determine the optimal strategies for the prevention and management of subsequent infection.

## Supporting Information

S1 File Liver donor and recipient characteristics, operation features characteristics and microorganisms isolated in blood, bile, ascites and preservation fluid cultures. (SAV)
